# Evaluation of the Radiotherapy Treatment Planning in the Presence of a Magnetic Valve Tissue Expander

**DOI:** 10.1371/journal.pone.0117548

**Published:** 2015-02-13

**Authors:** Débora M. Trombetta, Simone C. Cardoso, Victor G. L. Alves, Alessandro Facure, Delano V. S. Batista, Ademir X. da Silva

**Affiliations:** 1 Nuclear Engineering Program/Alberto Luiz Coimbra Institute for Graduate Studies and Research in Engineering (COPPE), Universidade Federal do Rio de Janeiro, Rio de Janeiro, Brazil; 2 Laboratório de Física da Radiação Gama—Instituto de Física, Universidade Federal do Rio de Janeiro, Rio de Janeiro, Brazil; 3 Serviço de Qualidade em Radiações Ionizantes—Instituto Nacional de Câncer, Rio de Janeiro, Brazil; 4 Comissão Nacional de Energia Nuclear, Rio de Janeiro, Brazil; 5 Instituto Nacional de Câncer—Seção de Física Médica, Rio de Janeiro, Brazil

## Abstract

The combination of radiotherapy treatments and breast reconstruction, using temporary tissue expanders, generates several concerns due to the presence of a magnetic valve inside the radiation field. The objective of this work is to evaluate a radiotherapy treatment planning for a patient using a tissue expander. Isodose curve maps, obtained using radiochromic films, were compared to the ones calculated with two different dose calculation algorithms of the Eclipse radiotherapy Treatment Planning System (TPS), considering the presence or absence of the heterogeneity. The TPS calculation considering the presence of the heterogeneity shows changes around 5% in the isodose curves when they were compared with the calculation without heterogeneity correction. This calculation did not take in account the real density value of the heterogeneity. This limitation was quantified to be around 10% in comparison with the TPS calculation and experimental measurements using the radiochromic film. These results show that the magnetic valve should be taken in account in dose calculations of the TPS. With respect to the AAA and Pencil Beam Convolution algorithms, when the calculation is compared with the real distribution, AAA presents a distribution more similar to experimental dose distribution.

## Introduction

The technique of breast reconstruction, that makes use of a temporary tissue expander, has been increased in the preference of mastectomized women. Among the benefits of this procedure, the short duration of the surgery, the mammary volume manipulation, preservation of sensibility and colour skin can be highlighted [1]. This technique is divided in two stages. First, the tissue expander (a silicon bag) is implanted totally empty above the breast muscle of the patient. Following, it is periodically inflated with a saline solution until it reaches the desired volume. In order to find and fill the expander inside the patient’s body, the manufacturer installs a magnetic valve is on the surface of the silicon bag and, using a magnetic locator, the valve can be found and the expander filled with saline solution injections. In a second stage, the tissue expander is removed and a permanent prosthesis is inserted in its place.

On the other hand, many trials have demonstrated that post-mastectomy radiotherapy induces benefits associated with patient survival [2–3]. The radiotherapy treatment is usually performed 4–8 weeks after the mastectomy surgery [4]. As the tissue expander, placed during the mastectomy surgery, can remain within the patient’s body for up to 8 weeks (depending on the desired breast volume), several patients are using the tissue expander when they are submitted to the radiotherapy treatment. Generally, the volume of the expander is adjusted during the treatment, and the second stage of the reconstruction surgery is usually performed at the end of the treatment [5].

The combination of radiotherapy and breast reconstruction generates several concerns in physicians and physicists due to the presence of the magnetic valve inside the radiation field. The effect of high atomic number materials on radiation fields have already been studied for many situations [6–16]. The expected effects are backscatter, rebuildup and attenuation of the beam, at the interface or after the high density material. Some authors have already investigated these effects for the presence of expander’s magnetic valve in radiation fields [17–20]. The results show a significant dose decrease when the radiation beam crosses the artifact, but they are only significant for distances smaller than 5 cm from the valve [20].

Dose enhancement at the close vinicity of the magnetic valve was described by some authors. Chatzigiannis et al [17] found 9% of dose enhancement at 2 mm from the magnet valve surface, but the effect was found to be negligible for distances higher than 1 cm. Thompson and Morgan [19] described a dose increase of 11% at the valve edge, but this effect decreases with distance and, after 5 mm, is insignificant. Radiotherapy treatment of patients using tissue expanders was studied for 6, 15 and 18 MV photon beams [6–17]. The highest dose attenuation reported in the literature was found for a 6 MV photon beam, while the highest dose enhancement was pronounced for beams with higher energies, due to the increased contribution of the pair production effect [16]. Damast et al [18] show that a magnetic valve attenuates a standard 6 MV photon beam by 22% and a 15 MV beam by 16%. Christos et al describe an underdosage area in the shadow of the magnetic valve, which ranges from 6–13% for 6 MV photon beams and negligible effects for 18 MV. Also, according to these authors, dose enhancements of 9% and 12% were found for 6 MV and 18 MV photon beams, respectively [17].

The objective of this work, instead of measuring the X-ray beam attenuation and backscattering, is to evaluate how the radiotherapy treatment planning is affected by the presence of the magnetic valve tissue expander in the radiation fields. With this aim, a breast phantom consisting of agar was developed to be used both for experimental measurements and to generate CT images, to be used in the Eclipse radiotherapy Treatment Planning System (TPS). Isodose curve maps obtained using radiochromic films were compared the isodoses calculated with two different TPS algorithms with and without heterogeneity correction for Pencil Beam Convolution (PBC) and Analytical Anisotropic Algorithm (AAA). Monte Carlo simulations were performed to accurately verify the influence of the artifact in dose calculations, since there is a limitation in the treatment planning system to make use of the correct artifact density.

## Materials and Methods

### The Breast Phantom

A phantom can be compared to a biological tissue through their radiological similarity. Parameters as density, atomic number or mass coefficient attenuation can be used.

Gelatin/agar phantoms are usually used to mimic high-water content tissues [21]. Agar is a mixture of the polysaccharide agarose and a heterogeneous mixture of agaropectin [22–23]. It is a gelatinous substance that in boiled water swells absorbing as much as twenty times its own weight of water, and when cooled, sets to a gel at concentrations as low as 0.5%. Advantages as the easy of material acquisition and manipulation, the possibility to manufacture arbitrary shapes and the low cost of material make this material interesting. In order to simulate the mastectomized breast, which is composed almost entirely of breast muscle (high water content tissue) [24], a gelatin/agar phantom was chosen.

The magnetic device was inserted in the breast phantom at 1.5 cm from its surface. The distance between the phantom surface and the magnetic device represents the thickness of the skin and pectoral muscle, just above the position where the expander is implanted.

### Phantom validation for calculation and experimental measurements

With the aim of validating the use of this new phantom composition, a regular phantom with 20 cm of side and 2 cm of thickness was built and parameters as Hounsfield Units (HU) and absorbed dose were measured and compared with the well known parameters for water.

A CT scan of the phantom was performed using the Siemens Somatom Emotion Duo CT Scanner [25]. In an area of 10 cm^2^ there were 7230 pixels and the calculated HU average was 5HU. Since the HU value accepted for water is between 0 and ±5 HU [26] and for muscle tissue 0–30 HU [27], the composition of the phantom was validated to be used in the TPS dose calculations.

Absorbed dose measurements were performed at 2.5 cm depth with an ionization chamber both in a phantom composed by solid water (PTW–RW3) [28] and in the regular agar phantom produced in this work. Five measurements for each point were performed and the average dose was calculated for both phantoms.

### Treatment Planning

The breast phantom was positioned above the thorax of a female Alderson Rando Phantom [29], at breast position, and several sets of CT images with 3mm of thickness. The set of CT images was exported for the Eclipse/Varian [30] TPS and a typical radiotherapy treatment for post-mastectomy was planned. A 6MV photon beam was used, with prescription dose of 5000 cGy, tangent opposite fields with 12x15cm^2^ size defined at the entrance surface.

**Fig 1:**
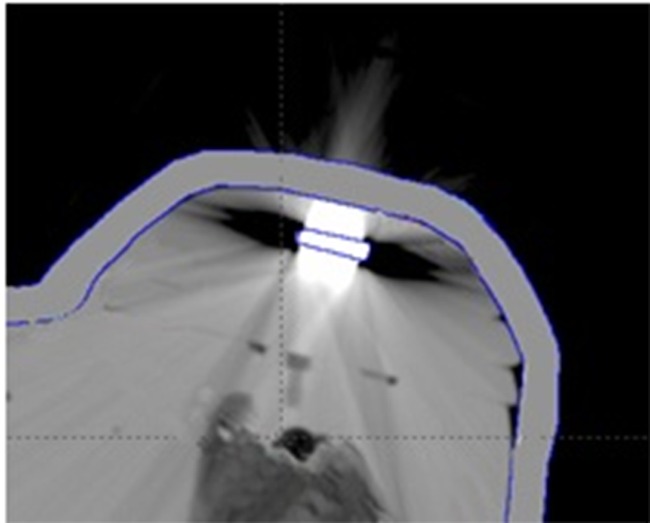
CT image showing artifacts due to the presence of the magnetic disk.

As expected, the magnetic material generated artifacts in the CT image, as illustrated in Fig. 1. Artifacts created zones of high and low densities around the device, modifying its real size, leading to an erroneous identification of the device by the treatment planning system. Thus, the device was manually identified in each image, as recommended by the TG 63 of AAPM [31]. Isodose curve maps were calculated using different approaches. Two dose calculation algorithms were used: Pencil Beam Convolution [32] and Analytic Anisotropic Algorithm [33], both with and without heterogeneity correction. For the calculation without heterogeneity correction, a density of 1 g/cm^3^ for all volume was assigned. For the calculations with heterogeneity correction, the density value was assigned to 5 g/cm^3^, that is the maximum limit that the treatment planning system can reach. However, the real artifact density is 7.4g/cm^3^ and this data could not be assigned in the TPS.

### Radiochromic Film Preparation and Irradiation Procedure

**Fig 2:**
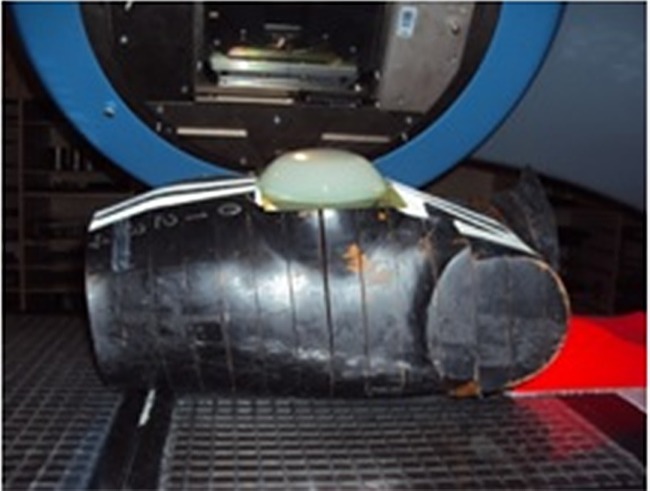
Radiochromic film positioned between Alderson Rando Phantom and agar breast phantom.

The radiochromic film Gafchromic EBT2 was manipulated according to the recommendations of the AAPM TG 55 [34]. The film was cut in pieces of 12x12cm. As the film is sensitive to the orientation when scanned, it was previously marked with a pen on the upper left corner [35]. One film piece was placed between the female Alderson Rando phantom thorax surface and the agar breast phantom, as shown in Fig. 2.

The set was irradiated with the same parameters used in the TPS calculations.

### Scanning Process and Image Analysis

The film was scanned in the first 24 h after the exposure, in order to avoid post exposure optical density changes. The scanner used was an EPSON 750V PRO, in transmission mode at 72 dpi and with a 48 bit RGB, depth of 16 bits per color channel, without applying any color correction, as recommended by the manufacturer [36]. The film analysis was performed according to the methodology developed by Alves et al [37].

### Monte Carlo Simulations

As the TPS did not allow the use of heterogeneity real density value, Monte Carlo simulations using the code MCNP were performed to verify the discrepancies that could exist due to this density difference. MCNP is a well-known general-purpose Monte Carlo N-particle code that can be used for neutron, photon, electron, or coupled neutron/photon/electron transport within an arbitrary three-dimensional configuration of materials in geometric cells; relating the created geometries with various classes of materials. A variety of sources can be defined by the user, from where electron, photon, and neutron emission are simulated, with probability distributions for energy and direction defined by the user. Subsequently, interactions are simulated according to the type of particle and material properties, as well as the production of secondary particles, which can be assessed for both particle fluence and energy deposition.

Computational calculation of absorbed doses has been accomplished for points situated at 1.5 cm above the device and at five depths under the artifact (1.5 cm, 2.5 cm, 3.5 cm, 4.5 cm and 5.5 cm). A simplified tungsten head shielding was simulated, using the 6MV energy spectrum presented by Rogers [38], and generating a field size of 10 x 10 cm^2^ at 100 cm, in the surface of the computational phantom.

## Results

### Breast phantom development

Three phantom compositions used for diagnostic purposes were tested with the respect to density and dimensional stability [24, 39,40].

None of them maintained their shape for more than 15 h at room temperature, despite having appropriate density values. An original phantom composed only of agar and water achieved the best set of results for density and dimensional stability. Using 15g of agar for each liter of water, a density of 1.06g/cm^3^ was obtained, and the weight, height and width, were maintained over 5 days at room temperature. Table 1 presents the results for the composition test.

To validate the agar phantom, the average dose was calculated to the phantom composed by solid water (PTW–RW3) [28] and in the regular agar phantom manufactured, Values obtained were the same, 0.102 cGy ± 1% (coefficient 30.998 cGy/nC). As the readings were equivalent, it was considered that the phantom is validated to be used for dose calculation in experimental measurements.

**Table 1:** Results for density and dimensional stability tests for some breast phantoms.

Author	Density	Dimensional Stability (T = 21°C)		
		Durability	Height/Width	Weight
Weinstein et al [39]	1.25 g/cm^3^	0h	5.0cm/5.0cm	156g
		5h	4.8cm/5.1cm	154g
		10h	4.6cm/5.2cm	152g
		15h	4.3cm/5.3cm	150g
Morehouse et al [24]	1.10 g/cm^3^	0h	5.0cm/5.0cm	137g
		5h	4.9cm/5.1cm	136g
		10h	4.7cm/5.1cm	134g
		15h	4.4cm/5.2cm	132g
Dang et al [40]	1.03 g/cm^3^	0h	5.0cm/5.0cm	129g
		5h	5.9cm/5.0cm	129g
		10h	4.9cm/5.0cm	128g
		15h	4.8cm/5.1cm	128g
Our phantom (86% agar solution)	1.06 g/cm^3^	0–15h	5.0cm/5.0cm	133g
		24h	4.9cm/4.9cm	133g
		48h	4.9cm/4.9cm	133g
		72h	4.9cm/4.9cm	133g

### Treatment Planning with heterogeneity corrections

**Fig 3:**
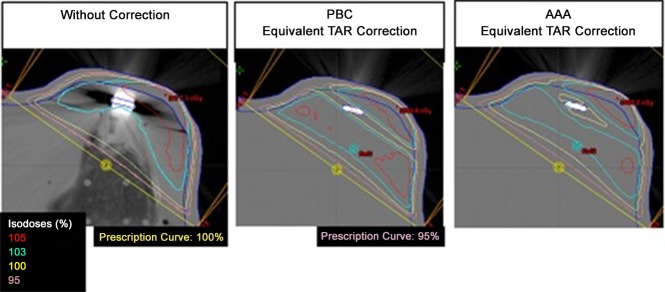
Maps of isodose curves calculated with and without correction for two different algorithms, PBC and AAA.

Comparisons between isodose curves maps calculated with and without the heterogeneity correction are shown in Fig. 3. When the heterogeneity correction was used, an underdosage narrow zone appears around the magnetic device. These differences are not greater than 5%. Comparing the two dose calculation algorithms applied, it can be observed that AAA shows a larger and more irregular isodose curve shape around the heterogeneity. Literature presents several studies comparing the two algorithms and the AAA has proved to be more accurate in dose calculations at heterogeneous media [41–43], therefore AAA was used to compare the TPS calculation with experimental measurements.

### TPS Calculation x Experimental Measurements

**Fig 4:**
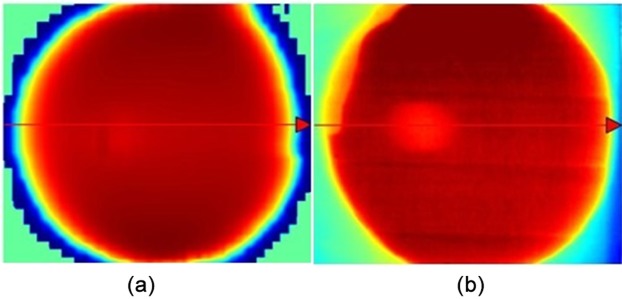
Isodose crossplane calculated with (a) TPS and (b) measured with radiochromic film.

Isodose crossplans calculated with the TPS and measured with radiochromic film were compared. As can be seen in Fig. 4, a circular light red area appears in both images, but it is clearer for the radiochromic film. The circular shape observed in the images reminds the circular shape of the magnetic device and it is suppose to be the cause of this underdosage area.

**Fig 5:**
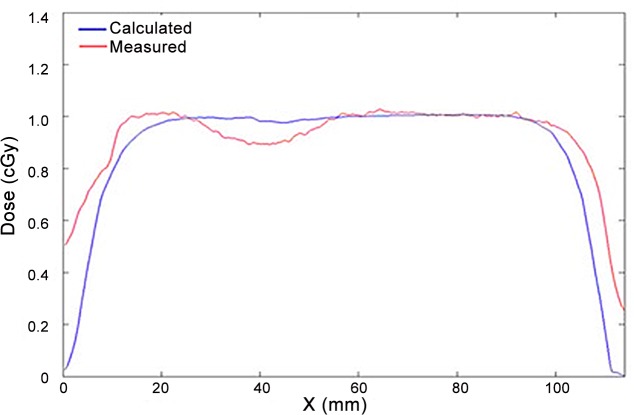
Transverse dose profile comparing the TPS and the radiochromic film reading.

A transverse dose profile was constructed to quantify this effect, as illustrated in Fig. 5. The profile from the TPS calculation shows a smooth decrease on the dose, less than 3%, whereas the profile from the film shows a steep decrease of almost 10%. This difference is statistically significant. The uncertainties related to experimental measurements and TPS calculations are smaller than 3%.

### Monte Carlo Simulations

**Fig 6:**
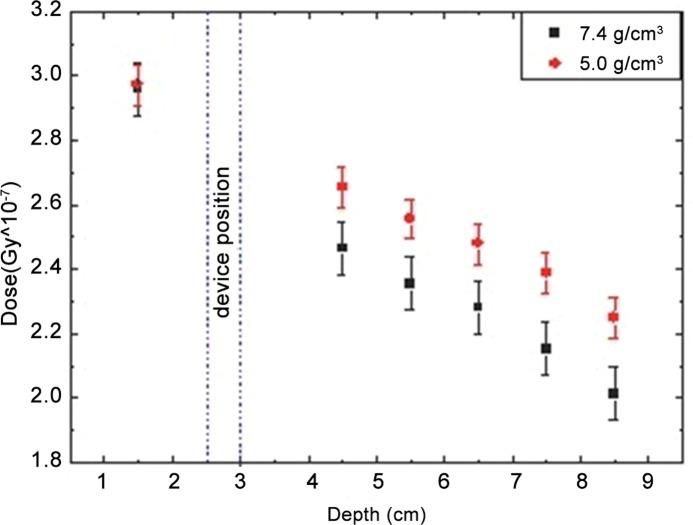
Dose x Depth Monte Carlo Simulations, comparing the two densities; black squares for 7.4g/cm^3^ and red circles for 5.0g/cm^3^.

The graphic presented in Fig. 6 shows the dose versus depth, calculated for the two values of artifact’s density:7.4 and 5.0 g/cm^3^. As can be seen, differences between the two densities lead to a discrepancy around 10% for the points beyond the device, in the tissue expander and chest wall location. Before the device, in the skin and breast tissue regions, the doses for both densities are the same.

As the results found with Monte Carlo simulations showed differences in attenuation calculations around 8–9% when the two densities were compared, the difference found between TPS and radiochromic film measurements was believed to be caused by the limitation of the TPS in assigning the nominal density value of the magnetic device.

## Discussion

### Treatment Planning with heterogeneity corrections

The presence of the magnetic device in the radiation field causes image artifacts, altering the CT number of the structures around it. These changes prevented the automatic discrimination by the TPS, and the solution was to employ the manual identification of the heterogeneity, as recommended by the AAPM TG-63. Damast [18] report the same difficulty in the TPS calculations due to the heterogeneity artifacts at the image. Thompson [19] also report difficulties in the identification of a magnetic valve in a CT image. The manual identification is not a practical procedure to be employed in the routine of a radiotherapy service, and another possible procedure could be minimizing artifacts using post-reconstruction algorithms [44, 45].

Dose calculations with heterogeneity corrections present differences around 5% when compared with calculations without heterogeneity corrections. Although the 5% value of isodose change obtained through TPS calculation apparently remains within the acceptable range [46], it must be noticed that this calculation did not consider the real density value of the heterogeneity, and the report AAPM-85 the precision of computational calculated doses should be around 1–2% [47].

The different isodose curves shapes found for AAA and PBC calculations is attributed to the fact that AAA is a 3D calculation algorithm, whereas PCB is 2D.

### TPS Calculation, Experimental Measurements and Monte Carlo Simulations

Radiochromic films results and Monte Carlo simulations show that the dose calculation by TPS with the limit of 5.0 g/cm^3^, could differ in the chest wall region about 10%, and it has to be emphasized that the chest wall is an important region to be treated. Thompson [19] also reported a limitation in the TPS used at her study, that makes use of a maximum density value of 2.83 g/cm^3^. This maximum value could be altered through the construction of a new calibration curve covering high densities values to the CT scanner/TPS set. This extension in the curve would be useful not only for the expander case treated here, but also for metallic prosthesis, as those made of Co-Cr-Mo (8.8 g/cm^3^).

## Conclusion

The composition used in the production of the agar breast phantom has proved to be an effective breast simulator, with density of 1.06 g/cm^3^. It could be used to obtain CT images and to perform experimental measurements. Furthermore, the developed agar solution has fast hardening (around 20 minutes), good resistance, and keeps its weight and shape up to 72 hours at room temperature.

Despite being cumbersome, the heterogeneity identification is very important, as the TPS calculation considering the presence of heterogeneity shows changes around 5% in the isodose curves when it was compared to calculation without heterogeneity correction. This limitation could be overcome through the calibration of the set CT scanner/TPS for high density materials. The significance of this limitation was quantified to be around 10% in comparison, beyond the device, with the TPS calculation and experimental measurements using the radiochromic film. This difference is only due the attenuation of the beam. Backscatter is not affected for the difference of densities evaluated. These results show that the magnetic valve must be taken in account in the dose calculations of the TPS.

With respect to the AAA and PBC algorithm, when the calculation is compared with the real distribution, AAA presents a distribution more similar to real dose distribution. And, as literature describes better results with AAA for heterogeneous media, it is the indicated algorithm inside TPS Eclipse Varian to this case.
